# Correction: Shared mechanisms between coronary heart disease and depression: findings from a large UK general population-based cohort

**DOI:** 10.1038/s41380-020-0857-7

**Published:** 2020-08-17

**Authors:** Golam M. Khandaker, Verena Zuber, Jessica M. B. Rees, Livia Carvalho, Amy M. Mason, Christopher N. Foley, Apostolos Gkatzionis, Peter B. Jones, Stephen Burgess

**Affiliations:** 1 Department of Psychiatry, University of Cambridge, Cambridge, UK; 2 Cambridgeshire and Peterborough NHS Foundation Trust, Cambridge, UK; 3 MRC Biostatistics Unit, University of Cambridge, Cambridge, UK; 4 Cardiovascular Epidemiology Unit, Department of Public Health and Primary Care, University of Cambridge, Cambridge, UK; 5 Department of Clinical Pharmacology, Queen Mary University of London, London, UK

**Keywords:** Depression, Genetics, Diagnostic markers, Diagnostic markers

Correction to: *Molecular Psychiatry* (2020) 25:1477-1486

10.1038/s41380-019-0395-3

Published online 19 March 2019

Following publication of this article, the authors became aware of an error regarding the effect alleles for genetic variants associated with circulating interleukin 6 (IL-6). The effect alleles for genetic variants associated with circulating interleukin 6 (IL-6) were misaligned due to an error propagated from a previous publication in which the minor allele of the IL6R variant rs7529229 was incorrectly labelled as the T allele [1].

Based on this error, effect alleles for three variants in the IL6R gene region require correction, which leads to changes in Supplementary Table 6, and Mendelian randomization estimates for IL-6.

The changes made to the original article are detailed below:

1. In the “Abstract”:

“The odds ratio for depression per standard deviation increase in genetically-predicted triglycerides was 1.18 (95% CI 1.09–1.27; *p* = 2 × 10^−5^); per unit increase in genetically-predicted log-transformed IL-6 was 0.74 (95% CI 0.62–0.89; *p* = 0.0012); and per unit increase in genetically-predicted log-transformed CRP was 1.18 (95% CI 1.07–1.29; *p* = 0.0009).”

was changed to:

“The odds ratio for depression per standard deviation increase in genetically-predicted triglycerides was 1.18 (95% CI 1.09–1.27; *p* = 2 × 10^−5^); per unit increase in genetically-predicted log-transformed IL-6 was 1.35 (95% CI 1.12–1.62; *p* = 0.0012); and per unit increase in genetically-predicted log-transformed CRP was 1.18 (95% CI 1.07–1.29; *p* = 0.0009).”

2. In the “Results” section, under the heading “Mendelian randomization analyses”:

“For the inflammatory biomarkers (Table 3), there was an evidence for causal effects of IL-6 and CRP, with an odds ratio of 0.74 (95% CI: 0.62–0.89, *p* = 0.0012) per unit increase in genetically-predicted values of log-transformed IL-6 (Fig. 1b)…”

was changed to:

“For the inflammatory biomarkers (Table 3), there was evidence for causal effects of IL-6 and CRP, with an odds ratio of 1.35 (95% CI 1.12–1.62; *p* = 0.0012) per unit increase in genetically-predicted values of log-transformed IL-6 (Fig. 1b)…”

Additionally, in this section:

“Although these results appear to be in different directions, variants in the IL6R gene region affect cellular binding of interleukin-6 receptor (IL-6R). Increased circulating concentrations of IL-6 do not represent increased production of IL-6, but rather reduced cellular binding [37]. Hence for both inflammatory markers, increased levels of the inflammatory marker lead to greater risk of depression.”

was changed to:

“The alleles associated with increased IL-6 levels are also associated with decreased CRP levels. This provides a discrepancy in the interpretation of results based on variants in the IL6R and CRP gene regions: variants in the CRP gene region associated with increased CRP levels were associated with increased risk of depression, whereas variants in the IL6R gene region associated with increased circulating IL-6 levels but decreased IL-6 activity and decreased CRP levels were associated with increased risk of depression. Further investigation is needed to understand this discrepancy, which could be linked to divergent effects of IL-6 classical and trans signalling on depression risk, and/or CRP-dependent vs CRP-independent effects of IL-6 on depression risk.”

3. In Table 3, in the row IL-6, the odds ratio (95% CI) was changed from “0.74 (0.62–0.89)” to “1.35 (1.12–1.62)”.

4. Figure 1b has been corrected. In the corrected figure, for each of the three variants in the IL6R gene region considered, the allele associated with increased circulating IL-6 concentrations is also associated with increased risk of depression. The correct Mendelian randomization estimate based on these genetic variants is an odds ratio of 1.35 (95% confidence interval 1.12–1.62, *p* = 0.0012) for depression per unit increase in log-transformed circulating levels of IL-6.

The original, incorrect version of Fig. 1 is displayed below for reference.


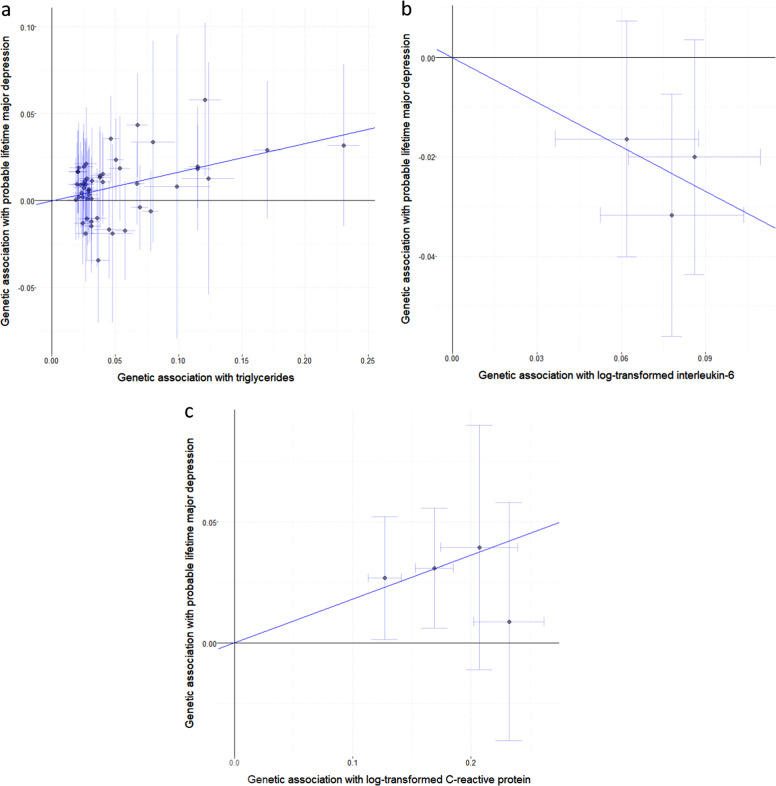


5. The supplementary file has been replaced. The changes made to this file are as follows:

In supplementary Table 6, the effect alleles for IL-6 biomarkers were corrected from “T, T, T”, to “C, C, G”.

In Supplementary Table 9, the odds ratio (95% CI) for IL-6 was changed from “0.74 (0.58 to 0.93)”, to “1.36 (1.07 to 1.72)”.

In Supplementary Table 10, the odds ratio (95% CI) for IL-6 was changed from “0.76 (0.58–1.00)”, to “1.32 (1.00–1.74)”.

The overall finding from the manuscript abstract that “IL-6, CRP and triglycerides, are likely to be causally linked with depression, so could be targets for treatment and prevention of depression” remains true, even though the direction of estimate for IL-6 was incorrect. The *p*-values for the associations of genetically-predicted IL-6 with depression remain unchanged.

This has been corrected in both the PDF and HTML versions of the article.
